# The Circadian Nature of Mitochondrial Biology

**DOI:** 10.3389/fendo.2016.00162

**Published:** 2016-12-19

**Authors:** Gal Manella, Gad Asher

**Affiliations:** ^1^Department of Biomolecular Sciences, Weizmann Institute of Science, Rehovot, Israel

**Keywords:** circadian rhythms, clocks, mitochondria, mitochondrial respiration, reactive oxygen species, oxygen, mitochondrial dynamics

## Abstract

Circadian clocks orchestrate the daily changes in physiology and behavior of light-sensitive organisms. These clocks measure about 24 h and tick in a self-sustained and cell-autonomous manner. Mounting evidence points toward a tight intertwining between circadian clocks and metabolism. Although various aspects of circadian control of metabolic functions have been extensively studied, our knowledge regarding circadian mitochondrial function is rudimentary. In this review, we will survey the current literature related to the circadian nature of mitochondrial biology: from mitochondrial omics studies (e.g., proteome, acetylome, and lipidome), through dissection of mitochondrial morphology, to analyses of mitochondrial processes such as nutrient utilization and respiration. We will describe potential mechanisms that are implicated in circadian regulation of mitochondrial functions in mammals and discuss the possibility of a mitochondrial-autonomous oscillator.

## Introduction

Light-sensitive organisms harbor molecular oscillators that measure time with periodicity of about a day known as circadian clocks. These clocks enable organisms to optimize a wide range of biological functions with the geophysical time (1–3). In mammals, these activities include rest/activity cycles, feeding/fasting, and various other physiological processes. The mammalian circadian timing system consists of a central pacemaker in the suprachiasmatic nucleus (SCN) of the brain that synchronizes subsidiary oscillators in the rest of the body. While the brain’s “master clock” is entrained by daily light–dark cycles, the dominant timing cue for clocks in peripheral organs appears to be feeding time. Circadian clocks are believed to function based on negative transcription–translation feedback loops generated through the action of several core clock genes. These include the transcriptional activators *Clock* and *Bmal1*, the repressors *Per1/2/3* and *Cry1/2*, and the nuclear receptors’ family members *Rev-Erb* and *Ror*. These clocks tick in virtually every cell of the body and function in a self-sustained and cell-autonomous manner.

Growing evidence support the presence of an intricate interplay between circadian clocks and metabolism. Circadian clocks play a prominent role in the regulation of various metabolic pathways. In turn, several metabolites and metabolic processes are implicated in the clock’s function. Several comprehensive reviews have covered in detail the molecular architecture of the core clock machinery (4–7) and their interplay with metabolism (2, 8–11). Among the large number of studies on circadian control of metabolism, only a handful of studies investigated in-depth circadian facets of mitochondrial function. Mitochondria constitute major metabolic hubs in eukaryotic cells involved in many vital processes including energy production via aerobic respiration, lipid biosynthesis, and calcium homeostasis. It is, therefore, conceivable that some of these functions might be under circadian clock control.

We review herein the current literature related to the circadian nature of mitochondrial biology in mammals. We elaborate on potential mechanisms underlying circadian control of mitochondrial functions and discuss the possibility of a mitochondrial-autonomous oscillator.

## Circadian Rhythms in Mitochondrial Composition

Rhythmic changes in the proteome, acetylome, and lipidome of mitochondria were uncovered lately as detailed below. These changes are expected to support rhythms in mitochondrial functions.

### The Mitochondrial Proteome

The mitochondrial proteome consists of several hundred different proteins (12). While the majority of the mitochondrial proteome is encoded by the nuclear genome (13) and transported into mitochondria via protein import machinery, only 13 protein-coding genes are transcribed and synthesized locally. Substantial daily changes in the mitochondrial protein composition were uncovered by whole liver proteomics (14, 15) and more recently by proteomic analyses of isolated mitochondria (16). In fact, over a third of the mitochondrial proteins accumulated in mitochondria in a daily manner (16). Notably, the vast majority of rhythmic proteins reached their zenith levels about the same time, during the early light phase. Further functional annotation of the rhythmic mitochondrial proteome evinced that key catabolic and oxidative functions of mitochondria exhibit diurnal oscillation (16). Of note, several components of the pyruvate dehydrogenase complex (PDC) that catalyzes the rate-limiting step in mitochondrial carbohydrate metabolism accumulate early in the light phase. While carnitine palmitoyl-transferase 1 (CPT1), the rate-limiting enzyme in the entry of fatty acids into the mitochondrial matrix, oscillates with zenith levels between the late dark and early light phase.

Both transcriptional and posttranscriptional events can potentially account for the above-described changes in the mitochondrial proteome. Indeed, the transcript levels of several nuclear-encoded mitochondrial proteins are altered in clock genes mutant mice (17, 18). Moreover, BMAL1 was shown to bind their promoters by ChIP (19, 20). However, global analysis evinced poor correlation between the phase of the mitochondrial proteome and its respective transcriptome (16). It is therefore likely that the observed daily changes in the mitochondrial proteome arise from posttranscriptional mechanisms such as rhythmic translation, protein import, and/or degradation. Future studies are expected to shed light on the contribution of these different mechanisms.

### The Mitochondrial Acetylome

Posttranslational modifications, such as phosphorylation, acetylation, and ubiquitinilation, control protein stability and activity. Global acetylome analysis of mouse liver identified daily changes in the acetylation status of many mitochondrial proteins (21). Remarkably, CLOCK-dependent acetylation sites were enriched for mitochondrial proteins including participants of the Krebs cycle and glutathione metabolism. Likewise, Peek and colleagues found that the acetylation status of many mitochondrial proteins differs between wild-type and BMAL1-deficient mice (22). For example, acetylation of fatty acid metabolism enzymes correlates with their activity and are BMAL1 dependant. In addition, the respiratory complex I is rhythmically acetylated, in accordance with changes in mitochondrial respiration (23). Overall, these results suggest that circadian clocks play a regulatory role in mitochondrial protein acetylation. It will be interesting to determine whether other posttranslational modifications of mitochondrial proteins, such as phosphorylation, are rhythmic as well, and further dissect their functional relevance.

### The Mitochondrial Lipidome

Lipids are the principal building blocks of biological membranes and among others define the physical qualities of mitochondrial membranes, as well as their protein content (24). In addition, lipids serve as a major energy source for mitochondrial respiration and some lipids are even synthesized in mitochondria. We recently applied high-throughput lipidomic analyses on isolated mitochondria from mouse liver to investigate the daily mitochondrial lipidome (25). We found that about one third of the lipids in mitochondria exhibit daily rhythms. Both the composition and phase of the rhythmic lipids depend on feeding regimen (nighttime restricted vs. *ad libitum* feeding) and circadian clock (PER1/2 null vs. wild type mice). In *ad libitum* fed mice, the majority of mitochondrial lipids reach their peak levels at the transition between the light and the dark phase, while an opposite phase is observed in mice fed exclusively during the dark phase. By contrast, in the absence of the core clock proteins PER1 and PER2, the oscillating lipids exhibit a wide range of peak times without an overt phase, supporting a role for circadian clocks in coordination of mitochondrial lipid accumulation (25). Likewise, mitochondrial fatty acid composition as well as their metabolism was reported to depend on BMAL1 (22). Future studies on these rhythmic lipids are expected to further clarify their relevance to the daily changes in mitochondrial morphology and function as detailed below.

## Circadian Rhythms in Mitochondrial Morphology

Mitochondrial dynamics, namely, changes in shape and size due to fission, fusion, and mitophagy, strongly affect mitochondrial function. In general, respiration is more efficient in fused mitochondria compared to fragmented mitochondria, primarily due to changes in nutrient availability (26).

Early electron microscopy works showed that the shape and volume of mitochondria change between the light and dark phase in rat hepatocytes (27). A recent study identified daily rhythms in mitochondrial dynamics in mouse liver and revealed that many genes participating in mitochondrial dynamics are expressed in a daily manner and are dependent on BMAL1 (20). Consequently, mitochondria isolated from *Bmal1* liver-specific knockout mice are bigger, more rounded, and do not exhibit morphological changes throughout the day. Additionally, in the absence of *Bmal1*, mitochondria are more susceptible to oxidative stress-related damage.

Dependency of mitochondrial morphology on clock genes was also reported in mouse skeletal muscle (28) and heart (17) and is linked to impaired mitochondrial function in these organs. Mitochondria in macrophages exhibit daily morphological changes *in vitro* as well (29). By contrast, the overall number of mitochondria, assessed by mitochondrial genome copy number, appears to be constant throughout the day and is independent of clock genes (16, 17, 20, 30). Collectively, these studies point to circadian regulation of mitochondrial dynamics, such as changes in mitochondrial mass and morphology, with major implications on mitochondrial function.

## Circadian Rhythms in Mitochondrial Nutrient Utilization and Respiration

A central function of mitochondria is energy production through nutrient oxidation, a process known as oxidative phosphorylation. Pyruvate and fatty acids are catabolized into acetyl CoA in the mitochondrial matrix through the action of the PDC and fatty acid oxidation (FAO), respectively. The acetyl groups are then fed into the Krebs cycle, and the process culminates with the transfer of acetyl-derived high-energy electrons along the respiratory chain. This process is coupled to production of ATP by the ATP synthase complex upon flux of protons through the inner mitochondrial membrane. In recent years, several studies tested the circadian control of mitochondrial nutrient utilization and respiration, using assays that measure oxygen consumption rate (OCR) in cultured cells and isolated mitochondria as detailed below. OCR measurements of synchronized C2C12 muscle cells in culture are rhythmic with ~24 h period (22). Similar results were obtained with HepG2 cells, albeit with a significantly shorter period (~15 h) (23). Analysis of isolated hepatocytes from wild-type mice harvested in different times of the day revealed higher respiration levels during the dark phase compared to the light phase in the presence of pyruvate. These daily differences were diminished in hepatocytes derived from liver-specific BMAL1-deficient mice (20).

Additional analyses of mitochondrial respiration were conducted with isolated mitochondria from mouse liver, muscle, and rat brain (16, 22, 28, 31). Mitochondria isolated from livers of wild-type mice exhibit higher OCR than those of *Bmal1* knockout mice (22), *Bmal1* liver-specific knockout mice (20), and *Per1/2* double knockout mice (16). Likewise, measurements of FAO by [^14^C]-labeled fatty acid supplementation evinced that this property is also reduced in *Bmal1* knockout mice (22). Experiments performed with mitochondria isolated from mice around the clock shed light on daily aspects of mitochondrial nutrient utilization. In the presence of succinate, the respiration of mitochondria is constant throughout the day (20) (Asher lab, unpublished data). By contrast, supplementation of FAO substrates such as palmitoyl-carnitine and palmitoyl-CoA + carnitine results in rhythmic respiration with zenith level early in the light phase, in accordance with CPT1 protein levels. Carbohydrates (i.e., pyruvate and malate) utilization is rhythmic as well, but peaks later during the light phase (16). The differences in peak time of mitochondrial respiration in experiments conducted with isolated mitochondria (16) vs. hepatocytes (20) might reflect the role of mitochondrial extrinsic cellular mechanism in controlling mitochondrial respiration. Remarkably, these daily rhythms in mitochondrial respiration are strongly influenced not only by the molecular circadian clock but also by nutrition type (e.g., high fat diet) and eating pattern (i.e., nighttime restricted feeding). Each of these factors differentially affects the overall level, rhythm, and phase of oscillation for several mitochondrial enzymes and the processing of their respective substrates (16).

Taken together, these studies suggest that mitochondrial respiration exhibits daily rhythms that are dependent on the molecular clock, nutrients, feeding pattern, and diet composition.

## Oxygen and Mitochondrial Rhythmicity

Reactive oxygen species (ROS) are byproducts of mitochondrial oxidative activity. The ROS hydrogen peroxide (H_2_O_2_) is scavenged by the peroxiredoxins (Prx’s) protein family members, which are reversibly oxidized to generate Prx-SOH. Upon high peroxide concentration, a hyperoxidized form of Prx, namely, Prx-SO_2_, accumulate and can then be reduced by sulfiredoxin (Srx). Prx3, the mitochondrial isoform of Prx, exhibits daily oscillations in its oxidation state. These oscillations are dependent on Srx levels in mitochondria, which are rhythmic as well and are regulated through its mitochondrial import and degradation (32). The regulation on Srx is ROS dependent and therefore generates a metabolic feedback loop between ROS levels Prx3 and Srx. Of note, the oscillations in Prx3-SO_2_ levels were shown to play an important role in rhythmic production of corticosterone from cholesterol in the adrenal gland mitochondria (33).

Oxygen is obligatory for mitochondrial aerobic respiration, and recent studies identified a reciprocal interplay between oxygen and the circadian clock (34–36). Rhythmic oxygen levels reset circadian clocks through HIF1α (34). Concomitantly, clock genes in concert with HIF1α regulate mitochondrial respiration upon changes in oxygen levels (35). Thus, both hands, namely, ROS and hypoxia, appear to intertwine with circadian control of mitochondrial function.

## The SCN Clock and Mitochondrial Function

Several studies examined mitochondrial activity of SCN neuron in the context of circadian clock function. Notably, SCN cells exhibit daily rhythms in cytochrome *c* oxidase activity and mitochondrial membrane potential (37). Moreover, studies with SCN astrocytes identified circadian oscillation in calcium release from mitochondria (38, 39). These rhythms have been linked to oscillations in extracellular ATP concentration, which appear to play a role in coupling of SCN neurons (38, 39). Furthermore, treatment of mice with the mitochondrial toxin 3-nitropropionic acid results in disruption of SCN clock outputs, as monitored by behavioral rhythms and *ex vivo* reporter measurements (40).

## Autonomous Mitochondrial Rhythms

Mitochondria are considered as successors of ancient aerobic bacteria, consumed by an early eukaryotic predator cell over 1.5 billion years ago (41). This theory, known as endosymbiotic theory, proposes that mitochondria were once independent free-living organisms and possessed the full machinery for survival and reproduction. However, throughout evolution, some of their capabilities have been lost or transferred to the host cell. It is possible that the mitochondrial ancestor has possessed some kind of an oscillator to temporally coordinate contradictory metabolic processes. As such, even the simplest mitochondria-possessing cells, i.e., the budding yeast, present oscillations in mitochondrial oxidative activity (42). The “yeast metabolic cycle” shares some conserved characteristics with the circadian clock (42, 43), although its period is only of few hours. Given the high conservation of mitochondrial rhythms from yeast to mammals, and the observed rhythms in ROS-related mitochondrial enzymes, it is tempting to speculate that mitochondria harbor their own autonomous clocks. Along this line, a self-sustained transcription-independent mitochondrial oscillator has been suggested in the form of Prx3-SO_2_ (32), yet so far, its independency from the transcription–translation-based clock has not been established.

## Summary and Open Questions

**Figure 1:**
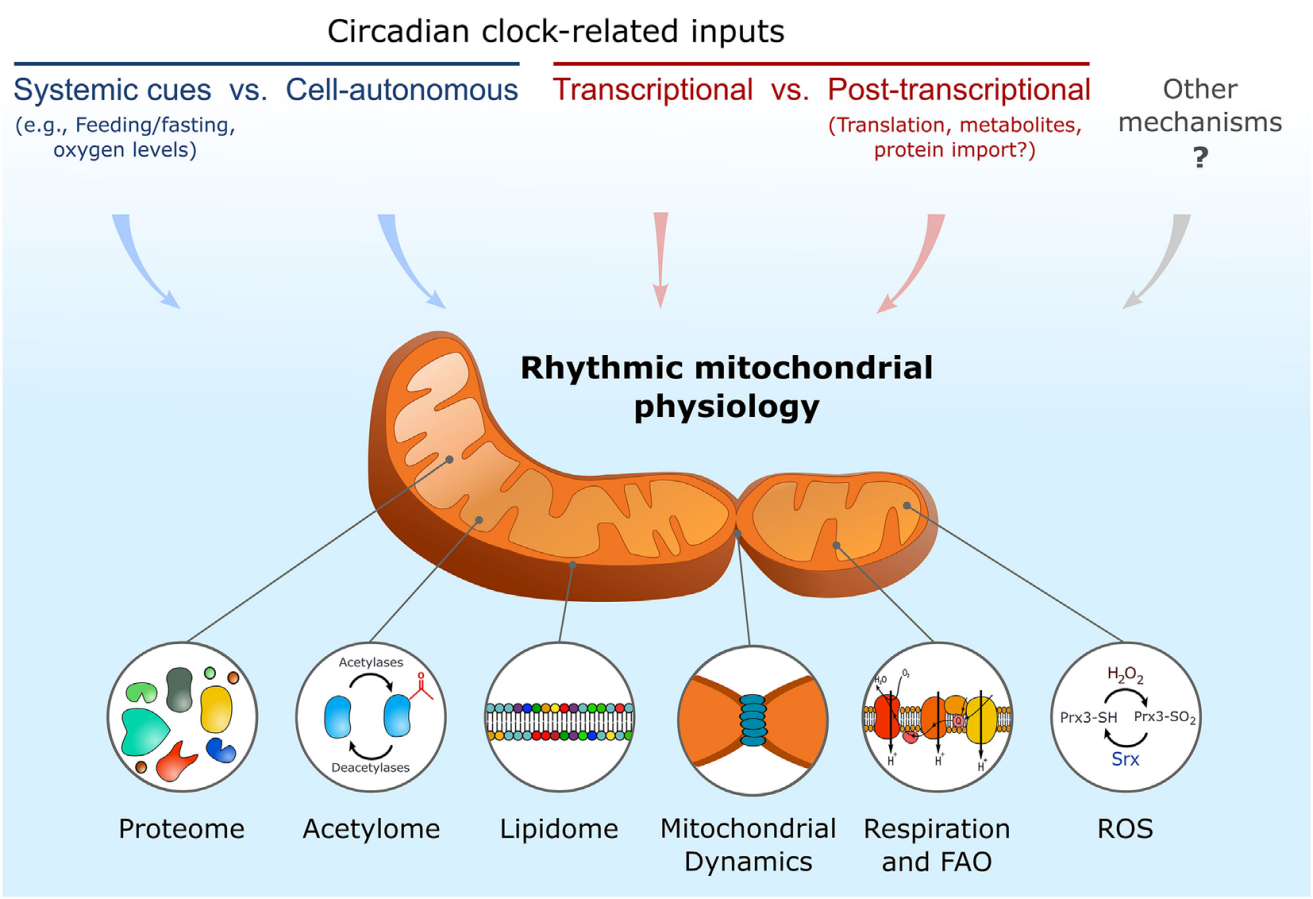
**Schematic depiction of key input mechanisms that participate in regulation of various circadian mitochondrial outputs**. ROS, reactive oxygen species; FAO, fatty acid oxidation.

In this review, we discussed daily rhythmicity in mitochondrial composition, morphology, and function alongside their underlying regulatory mechanisms (Figure 1). As shown, experiments performed with different clock mutant models support the potential role of circadian clocks in control of mitochondrial rhythmicity. However, it cannot be excluded that some of these effects are attributed to specific clock genes irrespective of their function within the core clock oscillator. In this respect, CRY proteins were reported to localize also in mitochondria; however, their specific mitochondrial function is unknown (44).

Another question is whether mitochondrial rhythmicity is achieved through systemic cues (such as feeding–fasting or rest–activity cycles) or via cell-autonomous mechanisms. It is likely that both scenarios co-regulate mitochondrial homeostasis throughout the day. In this conjuncture, experiments addressing mitochondrial function in cultured cells support a cell-autonomous effect on mitochondrial function. Whereas experiments with mice show that feeding rhythms are sufficient to restore some mitochondrial functions even in the absence of a functional clock. Remarkably, the ability of mitochondria to preserve functional differences when isolated in different hours of the day indicates that these alterations are not simply because of daily changes in substrate availability, but rather due to an inherent change in mitochondrial composition.

The lack of phase correlation between the mitochondrial proteome and its respective transcriptome (16) is another puzzling point. This finding highlights the importance of posttranscriptional mechanisms in control of mitochondrial protein homeostasis throughout the day. Recently, translation efficiency was reported to exhibit daily rhythms, specifically with respect to genes implicated in mitochondrial function (45, 46). An additional potential mechanism involves rhythmic regulation of protein import into mitochondria. Mitochondrial import machinery consists of several membrane protein complexes, such as the translocase of the outer (TOM) and translocase of the inner (TIM) mitochondrial membranes. Many subunits of TOM and TIM complexes are rhythmic in mitochondria (16). Moreover, the assembly of these complexes is regulated by several kinases, including casein kinase (CK) 1 and 2 (47, 48), which are widely recognized as regulators of circadian rhythmicity (49). Interestingly, cardiolipin lipids that are known to stabilize the import protein complexes (24) are also rhythmic in mitochondria isolated from mouse liver (25, 50). Given that the majority of mitochondrial proteins accumulate early in the light phase (16), it is presumable that the import machinery is gated to this time of the day and therefore dictate the daily changes in the mitochondrial proteome and function.

Bass and colleagues (22) proposed another model wherein circadian clocks generate oscillations in NAD^+^ levels, a cofactor for Sirtuin, a family of NAD^+^-dependent deacetylases, among them the mitochondrial SIRT3. Thus, NAD^+^ serves as a metabolic link between circadian clocks and mitochondrial function through NAD^+^ and SIRT3-dependent deactylation. In support of this model, they reported that reduction in mitochondrial activity in the absence of BMAL1 could be rescued by restoring NAD^+^ levels.

Future studies are expected to shed light on many of these and other open questions that are related to the circadian nature of mitochondrial biology.

## Author Contributions

GM and GA wrote the manuscript together.

## Conflict of Interest Statement

The authors declare that the research was conducted in the absence of any commercial or financial relationships that could be construed as a potential conflict of interest.

## References

[1] Asher G Schibler U. Crosstalk between components of circadian and metabolic cycles in mammals. Cell Metab (2011) 13(2):125–37. 10.1016/j.cmet.2011.01.006 21284980

[2] Bass J Circadian topology of metabolism. Nature (2012) 491(7424):348–56. 10.1038/nature11704 23151577

[3] Mohawk JA Green CB Takahashi JS. Central and peripheral circadian clocks in mammals. Annu Rev Neurosci (2012) 35:445–62. 10.1146/annurev-neuro-060909-153128 22483041 PMC3710582

[4] Dardente H Cermakian N. Molecular circadian rhythms in central and peripheral clocks in mammals. Chronobiol Int (2007) 24(2):195–213. 10.1080/07420520701283693 17453843

[5] Feng D Lazar MA. Clocks, metabolism, and the epigenome. Mol Cell (2012) 47(2):158–67. 10.1016/j.molcel.2012.06.026 22841001 PMC3408602

[6] Partch CL Green CB Takahashi JS. Molecular architecture of the mammalian circadian clock. Trends Cell Biol (2014) 24(2):90–9. 10.1016/j.tcb.2013.07.002 23916625 PMC3946763

[7] Dibner C Schibler U Circadian timing of metabolism in animal models and humans. J Intern Med (2015) 277:513–27. 10.1111/joim.12347 25599827

[8] Marcheva B Ramsey KM Peek CB Affinati A Maury E Bass J Circadian clocks and metabolism. Handb Exp Pharmacol (2013) 217:127–55. 10.1007/978-3-642-25950-0_6 PMC4089089 23604478

[9] Bailey SM Udoh US Young ME. Circadian regulation of metabolism. J Endocrinol (2014) 222(2):R75–96. 10.1530/JOE-14-0200 24928941 PMC4109003

[10] Asher G Sassone-Corsi P. Time for food: the intimate interplay between nutrition, metabolism, and the circadian clock. Cell (2015) 161(1):84–92. 10.1016/j.cell.2015.03.015 25815987

[11] Reinke H Asher G. Circadian clock control of liver metabolic functions. Gastroenterology (2016) 150(3):574–80. 10.1053/j.gastro.2015.11.043 26657326

[12] Cotter D Guda P Fahy E Subramaniam S. MitoProteome: mitochondrial protein sequence database and annotation system. Nucleic Acids Res (2004) 32(Database issue):D463–7. 10.1093/nar/gkh048 14681458 PMC308782

[13] Taanman J-W. The mitochondrial genome: structure, transcription, translation and replication. Biochim Biophys Acta (1999) 1410(2):103–23. 10.1016/S0005-2728(98)00161-3 10076021

[14] Mauvoisin D Wang J Jouffe C Martin E Atger F Waridel P Circadian clock-dependent and -independent rhythmic proteomes implement distinct diurnal functions in mouse liver. Proc Natl Acad Sci U S A (2014) 111(1):167–72. 10.1073/pnas.1314066111 24344304 PMC3890886

[15] Robles MS Cox J Mann M. In-vivo quantitative proteomics reveals a key contribution of post-transcriptional mechanisms to the circadian regulation of liver metabolism. PLoS Genet (2014) 10(1):e1004047. 10.1371/journal.pgen.1004047 24391516 PMC3879213

[16] Neufeld-Cohen A Robles MS Aviram R Manella G Adamovich Y Ladeuix B Circadian control of oscillations in mitochondrial rate-limiting enzymes and nutrient utilization by PERIOD proteins. Proc Natl Acad Sci U S A (2016) 113(12):E1673–82. 10.1073/pnas.1519650113 26862173 PMC4812734

[17] Kohsaka A Das P Hashimoto I Nakao T Deguchi Y Gouraud SS The circadian clock maintains cardiac function by regulating mitochondrial metabolism in mice. PLoS One (2014) 9(11):e112811. 10.1371/journal.pone.0112811 25389966 PMC4229239

[18] Gong C Li C Qi X Song Z Wu J Hughes ME The daily rhythms of mitochondrial gene expression and oxidative stress regulation are altered by aging in the mouse liver. Chronobiol Int (2015) 32(9):1254–63. 10.3109/07420528.2015.1085388 26512910

[19] Koike N Yoo SH Huang HC Kumar V Lee C Kim TK Transcriptional architecture and chromatin landscape of the core circadian clock in mammals. Science (2012) 338(6105):349–54. 10.1126/science.1226339 22936566 PMC3694775

[20] Jacobi D Liu S Burkewitz K Kory N Knudsen NH Alexander RK Hepatic Bmal1 regulates rhythmic mitochondrial dynamics and promotes metabolic fitness. Cell Metab (2015) 22(4):709–20. 10.1016/j.cmet.2015.08.006 26365180 PMC4598294

[21] Masri S Patel VR Eckel-Mahan KL Peleg S Forne I Ladurner AG Circadian acetylome reveals regulation of mitochondrial metabolic pathways. Proc Natl Acad Sci U S A (2013) 110(9):3339–44. 10.1073/pnas.1217632110 23341599 PMC3587221

[22] Peek CB Affinati AH Ramsey KM Kuo HY Yu W Sena LA Circadian clock NAD+ cycle drives mitochondrial oxidative metabolism in mice. Science (2013) 342(6158):1243417. 10.1126/science.1243417 24051248 PMC3963134

[23] Cela O Scrima R Pazienza V Merla G Benegiamo G Augello B Clock genes-dependent acetylation of complex I sets rhythmic activity of mitochondrial OxPhos. Biochim Biophys Acta (2016) 1863(4):596–606. 10.1016/j.bbamcr.2015.12.018 26732296

[24] Osman C Voelker DR Langer T. Making heads or tails of phospholipids in mitochondria. J Cell Biol (2011) 192(1):7–16. 10.1083/jcb.201006159 21220505 PMC3019561

[25] Aviram R Manella G Kopelman N Neufeld-Cohen A Zwighaft Z Elimelech M Lipidomics analyses reveal temporal and spatial lipid organization and uncover daily oscillations in intracellular organelles. Mol Cell (2016) 62(4):636–48. 10.1016/j.molcel.2016.04.002 27161994

[26] Wai T Langer T Mitochondrial dynamics and metabolic regulation. Trends Endocrinol Metab (2016) 27(2):105–17. 10.1016/j.tem.2015.12.001 26754340

[27] Uchiyama Y. Circadian alterations in tubular structures on the outer mitochondrial membrane of rat hepatocytes. Cell Tissue Res (1981) 214(3):519–27. 10.1007/BF00233492 7214464

[28] Andrews JL Zhang X McCarthy JJ McDearmon EL Hornberger TA Russell B CLOCK and BMAL1 regulate MyoD and are necessary for maintenance of skeletal muscle phenotype and function. Proc Natl Acad Sci U S A (2010) 107(44):19090–5. 10.1073/pnas.1014523107 20956306 PMC2973897

[29] Oliva-Ramirez J Moreno-Altamirano MM Pineda-Olvera B Cauich-Sanchez P Sanchez-Garcia FJ. Crosstalk between circadian rhythmicity, mitochondrial dynamics and macrophage bactericidal activity. Immunology (2014) 143(3):490–7. 10.1111/imm.12329 24903615 PMC4212961

[30] Magnone MC Langmesser S Bezdek AC Tallone T Rusconi S Albrecht U The mammalian circadian clock gene Per2 modulates cell death in response to oxidative stress. Front Neurol (2014) 5:289 10.3389/fneur.2014.00289 25628599 PMC4292776

[31] Simon N Papa K Vidal J Boulamery A Bruguerolle B. Circadian rhythms of oxidative phosphorylation: effects of rotenone and melatonin on isolated rat brain mitochondria. Chronobiol Int (2003) 20(3):451–61. 10.1081/CBI-120021385 12868540

[32] Kil IS Ryu KW Lee SK Kim JY Chu SY Kim JH Circadian oscillation of sulfiredoxin in the mitochondria. Mol Cell (2015) 59(4):651–63. 10.1016/j.molcel.2015.06.031 26236015

[33] Kil IS Lee SK Ryu KW Woo HA Hu MC Bae SH Feedback control of adrenal steroidogenesis via H2O2-dependent, reversible inactivation of peroxiredoxin III in mitochondria. Mol Cell (2012) 46(5):584–94. 10.1016/j.molcel.2012.05.030 22681886

[34] Adamovich Y Ladeuix B Golik M Koeners MP Asher G. Rhythmic oxygen levels reset circadian clocks through HIF1α. Cell Metab (2016). 10.1016/j.cmet.2016.09.014 27773695

[35] Peek CB Levine DC Cedernaes J Taguchi A Kobayashi Y Tsai SJ Circadian clock interaction with HIF1α mediates oxygenic metabolism and anaerobic glycolysis in skeletal muscle. Cell Metab (2016). 10.1016/j.cmet.2016.09.010 27773696 PMC5226863

[36] Wu Y Tang D Liu N Xiong W Huang H Li Y Reciprocal regulation between the circadian clock and hypoxia signaling at the genome level in mammals. Cell Metab (2016). 10.1016/j.cmet.2016.09.009 27773697

[37] Isobe Y Hida H Nishino H. Circadian rhythm of metabolic oscillation in suprachiasmatic nucleus depends on the mitochondrial oxidation state, reflected by cytochrome c oxidase and lactate dehydrogenase. J Neurosci Res (2011) 89(6):929–35. 10.1002/jnr.22609 21416482

[38] Burkeen JF Womac AD Earnest DJ Zoran MJ. Mitochondrial calcium signaling mediates rhythmic extracellular ATP accumulation in suprachiasmatic nucleus astrocytes. J Neurosci (2011) 31(23):8432–40. 10.1523/JNEUROSCI.6576-10.2011 21653847 PMC3125703

[39] Marpegan L Swanstrom AE Chung K Simon T Haydon PG Khan SK Circadian regulation of ATP release in astrocytes. J Neurosci (2011) 31(23):8342–50. 10.1523/JNEUROSCI.6537-10.2011 21653839 PMC3135876

[40] Kudo T Loh DH Tahara Y Truong D Hernandez-Echeagaray E Colwell CS Circadian dysfunction in response to in vivo treatment with the mitochondrial toxin 3-nitropropionic acid. ASN Neuro (2014) 6(1):e00133 10.1042/AN20130042 24328694 PMC3891360

[41] Dyall SD Brown MT Johnson PJ. Ancient invasions: from endosymbionts to organelles. Science (2004) 304(5668):253. 10.1126/science.1094884 15073369

[42] Tu BP Kudlicki A Rowicka M McKnight SL. Logic of the yeast metabolic cycle: temporal compartmentalization of cellular processes. Science (2005) 310(5751):1152–8. 10.1126/science.1120499 16254148

[43] Causton HC Feeney KA Ziegler CA O’Neill JS. Metabolic cycles in yeast share features conserved among circadian rhythms. Curr Biol (2015) 25(8):1056–62. 10.1016/j.cub.2015.02.035 25866393 PMC4406945

[44] Kobayashi K Kanno S Smit B van der Horst GT Takao M Yasui A. Characterization of photolyase/blue-light receptor homologs in mouse and human cells. Nucleic Acids Res (1998) 26(22):5086–92. 10.1093/nar/26.22.5086 9801304 PMC147960

[45] Atger F Gobet C Marquis J Martin E Wang J Weger B Circadian and feeding rhythms differentially affect rhythmic mRNA transcription and translation in mouse liver. Proc Natl Acad Sci U S A (2015) 112(47):E6579–88. 10.1073/pnas.1515308112 26554015 PMC4664316

[46] Janich P Arpat AB Castelo-Szekely V Lopes M Gatfield D. Ribosome profiling reveals the rhythmic liver translatome and circadian clock regulation by upstream open reading frames. Genome Res (2015) 25(12):1848–59. 10.1101/gr.195404.115 26486724 PMC4665006

[47] Gallego M Virshup DM. Post-translational modifications regulate the ticking of the circadian clock. Nat Rev Mol Cell Biol (2007) 8(2):139–48. 10.1038/nrm2106 17245414

[48] Harbauer AB Zahedi RP Sickmann A Pfanner N Meisinger C. The protein import machinery of mitochondria – a regulatory hub in metabolism, stress, and disease. Cell Metab (2014) 19(3):357–72. 10.1016/j.cmet.2014.01.010 24561263

[49] Reischl S Kramer A. Kinases and phosphatases in the mammalian circadian clock. FEBS Lett (2011) 585(10):1393–9. 10.1016/j.febslet.2011.02.038 21376720

[50] Adamovich Y Rousso-Noori L Zwighaft Z Neufeld-Cohen A Golik M Kraut-Cohen J Circadian clocks and feeding time regulate the oscillations and levels of hepatic triglycerides. Cell Metab (2014) 19(2):319–30. 10.1016/j.cmet.2013.12.016 24506873 PMC4261230

